# Comparative study of neuropharmacological, analgesic properties and phenolic profile of Ajwah, Safawy and Sukkari cultivars of date palm (*Phoenix dactylifera*)

**DOI:** 10.1007/s13596-016-0239-5

**Published:** 2016-08-16

**Authors:** Bassem Yousef Sheikh, S. M. Neamul Kabir Zihad, Nazifa Sifat, Shaikh J. Uddin, Jamil A. Shilpi, Omer A. A. Hamdi, Hemayet Hossain, Razina Rouf, Ismet Ara Jahan

**Affiliations:** 1 College of Medicine, Taibah University, PO Box 456, Almadinah Almunawarah, 41411 Saudi Arabia; 2 Pharmacy Discipline, Life Science School, Khulna University, Khulna, 9208 Bangladesh; 3 Department of Chemistry, Faculty of Science and Technology, Alneelain University, 11121 Khartoum, Sudan; 4 BCSIR Laboratories, Bangladesh Council of Scientific and Industrial Research (BCSIR), Dhaka, 1205 Bangladesh

**Keywords:** Date palm, Open field test, Hole board test, (+)-catechin, (−)-epicatechin, *Trans*-ferulic acid

## Abstract

In addition to the rich nutritional value, date palm is also used in various ethnobotanical practices for the treatment of various disease conditions. Present investigation was undertaken to examine the neuropharmacological and antinociceptive effect of the ethanol extract of three date cultivars growing in Saudi Arabia, namely Ajwah, Safawy and Sukkari. Neuropharmacological effect was observed by pentobarbitone induced sleeping time, open field, and hole board test. Antinociceptive activity was tested by acetic acid induced writhing and hot plate test. The date extracts were also subjected to HPLC analysis to detect the presence of common bioactive polyphenols. All the three date extracts extended the pentobarbitone induced sleeping time, reduced locomotor activity in open field test and reduced exploratory behaviour in hole board test in mice. The extracts also reduced acetic acid induced writhing and delayed response time in hot plate test. The activities were stronger for Ajwah than the other two date cultivars. HPLC analysis indicated the presence of *trans*-ferulic acid in all three cultivars, while (+)-catechin and (−)-epicatechin only in Ajwah and Safawy. The observed neuropharmacological and analgesic activity could be partly due to the presence of (+)-catechin, (−)-epicatechin and *trans*-ferulic acid, three important plant polyphenols well known for their neuroprotective activity and their ability to exert antioxidant activity on brain cells. Present investigation also supports the ethnobotanical use of date palm to provide ameliorating effects in pain and CNS disorders.

**Electronic supplementary material:**

The online version of this article (doi:10.1007/s13596-016-0239-5) contains supplementary material, which is available to authorized users.

## Introduction

The ripe fruits of *Phoenix dactylifera* L. (Arecaceae), also known as date palm, plays an important role in social and economic perspective of the people living in the oasis of the Middle East by the virtue of its nutritional and pharmacological properties (Baliga et al. 2011). The fruit serves as an important source of nutrition in an arid region hostile to habitation of plants. It is believed that the date palm originated in the Middle East. Due to its rich food value, date was later naturalised in many parts of the world, and at present more than 2000 cultivars of *P. dactylifera* are known to grow around the globe (Guido et al. 2011). Apart from its use as a staple food, date palm enjoys its use in the ethnomedicinal practice for a wide range of ailments. Date palm is used for the treatment of liver disorders (Gill 1992), diabetes (Ziyyat et al. 1997), constipation, diarrhoea (Hmamouchi 1999), and as an aphrodisiac (Zaid and Arias-Jiménez 2002). Date fruits are taken alone or in combination with other ingredients to get relief from asthma (Zaid and Arias-Jiménez 2002), to reduce wrinkling of the skin (Bauza et al. 2001), as an expectorant and ameliorating in cough, bronchitis, respiratory disorders, to alleviate headache, to treat sexual debility and to increase immunity (Selvam 2008; Zaid and Arias-Jiménez 2002). Investigations revealed that date palm possesses antioxidant, antimutagenic (Vayalil 2002), antihaemolytic (Abuharfeil et al. 1999), antiviral (Jassim and Naji 2010), antifungal (Shraideh et al. 1998), anti-inflammatory (Mohamed and Al-Okabi 2004), antihyperlipidemic (Al-Maiman 2005), hepatoprotective (Al-Qarawi et al. 2004; Sheikh et al. 2014), nephroprotective (Al-Qarawi et al. 2008), gastroprotective (Al-Qarawi et al. 2005), anticancer (Ishurd and Kennedy 2005), immunostimulating (Puri et al. 2000), and gonadotropic (El-Mougy et al. 1991) activity. The date fruit is also rich in pharmacologically important phytochemical constituents including simple pheolics (*p*-hydroxy benzoic acid, protocatechuic acid, gallic acid, vanillic acid, syringic acid), phenylpropanoids (cinnamic acid, caffeic acid, *o*-caffeoyl shikimic acid, ferulic acid, sinapic acid, *o*-coumaric acid, *p*-coumaric acid) (Mansouri et al. 2005), carotenoids (β-carotene, lutein), sterols (cholesterol, campesterol, stigmasterol, β-sitosterol, isofucosterol) (Kikuchi and Miki 1978), flavonoids and their glycosides (catechin, *epi*-catechin, quercetin, luteolin, apigenin) (Hong et al. 2006), procyaninidins (Hong et al. 2006), and anthocyanins (Al-Farsi et al. 2005).

The Sukkari date is the best-selling date in Saudi Arabia. These golden-brown dates have patches of lighter colour and are medium or small cone shaped with a firm exterior. This date is characteristically sweet as compared to other cultivars with its chewy flesh. It grows mainly in Qassim, Saudi Arabia. Safawy is another popular date cultivar growing in Almadinah Almunawarah, Saudi Arabia. Safawy is oval shaped soft, moist variety of dates with dark brown texture. Unlike other dates, Ajwah dates are relatively smaller in size. Ajwah is round shaped, soft, dark brown coloured date which looks almost black with fine texture and white wrinkles. Ajwah has special interest to Muslims as it has been mentioned in the Prophetic medicine.

In Ayurveda date palm is known as *Kharjura* and is indicated for the treatment of psychosis, anxiety, cognitive dysfunction and many of the nervous system disorders (Shanmugapriya and Patwardhan 2012). The fruit is also used alone or in combination to treat sciatica, headache, hemicranias, and applied externally for inflammatory conditions including abscess, boils and ulcers (Shanmugapriya and Patwardhan 2012). Literature survey on date palm revealed that some Chinese and Japanese patented herbal preparations containing date palm as one of the component can be beneficial in treating sleeping disorders ( Katsumichi et al. 1997; Tian 2014). Furthermore, acute toxicity study with date palm extract prior to our project on biological investigation of date extracts revealed extended period of sleep in test animals. All these observations prompted us, as a part of our research on Prophetic medicine (El-Ameen et al. 2015; Halabi and Sheikh 2014; Maulidiani et al. 2015; Taha et al. 2015), to evaluate and compare neuropharmacological effects of two date cultivars growing in Madinah (Ajwah and Safawy) and one growing outside Madinah but within Saudi Arabia (Sukkari).

## Materials and methods

### Plant material and extraction

**Fig. 1:**
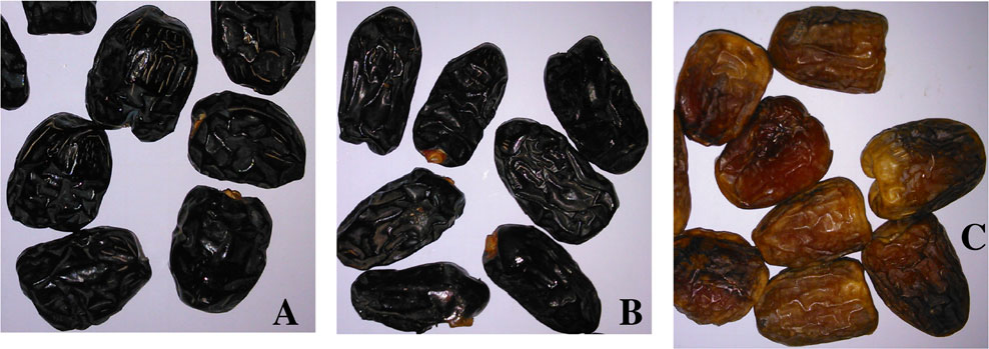
Pictures of date palms. **a**: Ajwah; **b**: Safawy; **c**: Sukkari

The dried ripe (in tamar stage) dates were purchased from local date market in Al Madinah AlMunawarah, Saudi Arabia. The dates were identified by taxonomists at Bangladesh National Herbarium where a voucher specimen (DACB 41158) has been submitted for future reference. For easy identification by the readers, images have been given in Fig. 1. The dried dates were mashed with the help of mortar and pestle, soaked in ethanol for 3 days with periodic sonication. The extracts were filtered and dried using a rotary vacuum evaporator at 45 °C under reduced pressure to get semisolid masses. The extracts were further freeze dried to get the crude extract.

### Test animals

Young Swiss Albino mice of 4–5 weeks old and weighing 20–25 g were purchased from the Animal Resources Branch of International Centre for Diarrhoeal Disease Research, Bangladesh (ICCDR,B). They were acclimatised with the laboratory condition (temperature: 25 ± 2 °C, relative humidity: 56–60 %, 12 h dark-light cycle) before the commencement of the pharmacological experiments.

### Chemicals and drugs

Arbutin, benzoic acid, caffeic acid, (+)-catechin, *trans*-cinnamic acid, *p*-coumaric acid, ellagic acid, (−)-epicatechin, *trans*-ferulic acid, gallic acid, hydroquinone, kaempferol, myricetin, quercetin, rosmarinic acid, rutin, syringic acid, vanillic acid, and vanillin were purchased from Sigma-Aldrich (St. Louis, MO, USA). Reference drugs were generously provided by Beximco Pharmaceuticals Ltd. Bangladesh (diclofenac sodium and caffeine), Popular Pharmaceuticals Ltd. Bangladesh (morphine) and Incepta Pharmaceuticals Ltd. Bangladesh (pentobarbitone).

### Acute toxicity test

Test mice divided into different groups containing 6 mice of either sex were treated with graded doses (62.5–4000 mg/kg body weight) of date palm extract while the control group received control vehicle (1 % Tween 80 in water) orally. The animals were observed for 72 h and mortality, general signs and symptoms of toxicity were recorded for each group (Lorke 1983).

### Grouping and dosing

Animals of either sex were randomly divided into four groups, each comprising of six animals. Control group received vehicle (1 % Tween 80 in water) orally at a volume of 10 ml/kg. Test groups were pre-treated orally with three date extracts at the doses of 250 and 500 mg/kg, while positive control group received reference drug.

### Pentobarbitone-induced sleeping time test

Test groups were orally treated with the three date extracts at the aforementioned doses while control and positive control group received control vehicle and diazepam (5 mg/kg, p.o.), respectively. After thirty minutes, pentobarbitone (50 mg/kg, i.p.) was administered to each mouse to induce sleep. The latent period for the onset of sleep, and the duration of sleep was recorded (Shilpi et al. 2004).

### Open field test

Test mice and control mice were placed on the floor of an open field of the dimension 100 cm × 100 cm × 40 cm, divided in squares coloured black and white. The number of squares visited by each group was recorded for 3 min after every thirty minutes starting from the time of extract administration and continued for a period of 4 h (Shilpi et al. 2004).

### Hole board test

Each mouse from the control, test and positive control group was placed in the hole board having 16 evenly placed holes. Head dipping of the mouse through the holes was recorded for 2 min on every 30 min for a period of 2 h starting from the time of extract administration (Mondal et al. 2014).

### Acetic acid induced writhing test

Acetic acid (0.7 %, 10 ml/kg) was administered intraperitoneally to each mouse 30 min after the administration of control vehicle, date extracts and diclofenac sodium (25 mg/kg, p.o.). After 5 min of acetic acid administration, number of writhing by each group was recorded for 10 min (Mondal et al. 2014).

### Hot-plate test

Control vehicle, date extracts and morphine (5 mg/kg, i.p.) treated mice were placed on a hot plate maintained at the temperature of 55 ± 0.5 °C on every 30 min starting from the time of extract administration and continued for a period of 2 h to record response time, i.e., the time required for paw licking or jumping. To avoid any injury or accidental paw damage of the mouse, a cut-off point of 15 s was maintained (Mondal et al. 2014).

### HPLC analysis for polyphenolic constituents

Detection of the major polyphenolic compounds present in the date extracts was conducted by HPLC analysis on a DionexUltiMate 3000 Rapid Separation LC system (Thermo Fisher Scientific Inc., MA, USA) equipped with a quaternary rapid separation pump (LPG-3400RS), acclaim® C_18_ column (4.6 × 250 mm; 5 μm, Dionex USA) housed in a temperature-controlled column compartment (TCC-3000) maintained at 30 °C, and photodiode array detector (DAD-3000RS) (Chuanphongpanich and Phanichphant 2006; Islam et al. 2014). Separation was done using a gradient elution programme consisting of 5%A95%B 0–9 min, 10%A80%B10%C 10–19 min, 20%A/60%B/20%C 20–30 min, followed by flushing and further equilibriation with 100%A for 5 min; where A, B and C are acetonitrile, acetic acid solution of pH 3 and methanol, respectively. For detection, photodiode array detector was set to the range of 200–700 nm for the entire experimental period while UV detector was set to 280 nm for 0–18 min, 320 nm for 19–24 min, and 380 nm for 25–30 min. Calibration curve was prepared using a standard solution of methanol containing arbutin (5 μg/ml), benzoic acid (8 μg/ml), caffeic acid (3 μg/ml), (+)-catechin (10 μg/ml), *trans*-cinnamic acid (1 μg/ml), *p*-coumaric acid (2 μg/ml), ellagic acid (10 μg/ml), (−)-epicatechin (5 μg/ml), *trans*-ferulic acid (3 μg/ml), gallic acid (4 μg/ml), hydroquinone (4 μg/ml), kaempferol (2 μg/ml), myricetin (4 μg/ml), quercetin (2 μg/ml), rosmarinic acid (4 μg/ml), rutin (6 μg/ml), syringic acid (3 μg/ml), vanillic acid (4 μg/ml), and vanillin (3 μg/ml). Test solutions for date extracts were prepared at a concentration of 5 mg/ml in methanol. The injection volume was 20 μl for standard or extract solutions, and the experiments were conducted with a flow rate of 1 ml/min.

### Statistical analysis

Results were expressed as mean ± SEM. One-way or two-way ANOVA followed by Bonferroni’s test was done for statistical analysis and results were considered significant when *p* < 0.05.

## Results

### Results of acute toxicity test

No mortality or signs or symptoms of toxicities were observed for any of the date extracts under investigation even at the highest dose (4.0 g/kg) tested. However, at higher doses, the mice showed extended sleeping tendency which persisted up to 48 h.

### Results of pentobarbitone-induced sleeping time test

All the date extracts reduced the time for the onset of sleep and increased the duration of sleep as compared to the control with the extent of intensity relatively higher with Ajwah than Safawy or Sukkari, but less than that of diazepam. All the results were statistically significant (Table 1).

**Table 1:** Effects of three date extracts on pentobarbitone induced sleeping time in mice

Treatment (*n* = 5)	Dose (mg/kg)	Route of administration	Onset of sleep (min)	Duration of sleep (min)
Control (1 % Tween 80 in water)	10 ml/kg	p.o.	9.6 ± 0.55	74.0 ± 2.0
Diazepam	5	i.p.	3.6 ± 0.34^d^	140 ± 2.2^f^
Ajwah	250	p.o.	7.8 ± 0.36^cd^	90 ± 2.4^ce^
	500	p.o.	6.3 ± 0.35^ad^	110 ± 2.2^cf^
Safawy	250	p.o.	8.1 ± 0.39^c^	86 ± 2.3^cd^
	500	p.o.	7.0 ± 0.35^cd^	100 ± 3.0^cf^
Sukkari	250	p.o.	8.6 ± 0.34^c^	83 ± 1.6
	500	p.o.	7.4 ± 0.24^cd^	97 ± 2.6^ce^

^a^ *p* < 0.05 vs. diazepam, ^b^ *p* < 0.01 vs. diazepam, ^c^ *p* < 0.001 vs. diazepam, ^d^ *p* < 0.05 vs. control, ^e^ *p* < 0.01 vs.control, ^f^ *p* < 0.001 vs. control

### Results of open field test

In the open field test, the extracts showed a decrease in the movements in test mice as compared to control. The decrease in the movement was prominent from 30 min until 120 min, with the effect gradually fading at 180 min. Diazepam, used as positive control showed similar results but the effect was stronger as compared to the three dates extracts. All the results were statistically significant (Table 2).

**Table 2:** Effects of three date extracts on open field test in mice

Treatment (*n* = 5)	Dose (mg/kg)	Number of movement					
		0 min	30 min	60 min	90 min	120 min	180 min
Control	10 ml/kg	133.2 ± 2.6	123.4 ± 3.23	113.0 ± 2.2	104.2 ± 2.3	106.8 ± 4.7	95.8 ± 2.0
Diazepam	5	126.2 ± 3.7	39.6 ± 1.7^f^	30.2 ± 1.0^f^	28.2 ± 2.5^f^	29.4 ± 1.2^f^	27.6 ± 1.2^d^
Ajwah	250	128.0 ± 2.0	89.4 ± 2.2^cf^	74.6 ± 2.7^cf^	73.2 ± 1.6^ce^	75.4 ± 2.8^cf^	81.0 ± 3.0^cd^
	500	129.2 ± 2.2	84.4 ± 1.8^cf^	71.6 ± 1.9^cf^	67.0 ± 1.4^cf^	71.0 ± 1.0^cf^	74.2 ± 1.7^cf^
Safawy	250	139.4 ± 2.6	91.6 ± 3.9^ce^	78.0 ± 3.1^cf^	76.0 ± 3.8^cf^	79.0 ± 2.5^ce^	84.4 ± 3.7^cd^
	500	133.0 ± 3.0	85.4 ± 1.9^cf^	73.4 ± 1.8^ce^	70.2 ± 1.9^cf^	75.2 ± 2.4^cf^	80.2 ± 3.8^cf^
Sukkari	250	129.4 ± 4.5	93.2 ± 2.8^cf^	82.2 ± 2.8^cf^	75.4 ± 2.7^ce^	73.0 ± 2.8^cf^	82.2 ± 1.9^ce^
	500	137.2 ± 3.6	86.2 ± 3.7^cf^	77.0 ± 3.3^cf^	72.4 ± 3.3^cf^	69.4 ± 2.7^cf^	79.2 ± 2.2^ce^

^a^ *p* < 0.05 vs. diazepam, ^b^ *p* < 0.01 vs. diazepam, ^c^ *p* < 0.001 vs. diazepam, ^d^ *p* < 0.05 vs. control, ^e^ *p* < 0.01 vs. control, ^f^ *p* < 0.001 vs. control

### Results of hole board test

In the hole board test, a decrease in the number of head dipping was observed for the test mice. Although, the effect was not as strong as that of diazepam, the results of the date extracts were significantly different when compared to control and the effect of Ajwah extract was stronger than the other two date extracts (Table 3).

**Table 3:** Effects of three date extracts on hole board test in mice

Treatment (*n* = 5)	Dose (mg/kg)	Number of head dipping					
		0 min	30 min	60 min	90 min	120 min	180 min
Control	10 ml/kg	19.2 ± 0.9	21.4 ± 1.3	27.0 ± 1.4	29.4 ± 1.6	31.0 ± 1.4	33.4 ± 1.3
Diazepam	5	20.2 ± 1.0	11.4 ± 0.9^f^	6.0 ± 1.0^f^	6.4 ± 0.8^f^	6.2 ± 0.6^f^	7.4 ± 0.5^f^
Ajwah	250	20.4 ± 0.8	16.8 ± 1.1^cd^	14.6 ± 0.8^cf^	13.8 ± 0.8^cf^	17.0 ± 0.7^ce^	23.8 ± 0.7^ce^
	500	20.4 ± 1.2	16.4 ± 1.2^be^	12.8 ± 0.9^cf^	12.4 ± 0.8^cf^	14.2 ± 1.0^cf^	19.8 ± 0.9^cf^
Safawy	250	19.8 ± 1.0	17.4 ± 0.9^cd^	18.0 ± 0.7^cf^	16.0 ± 0.5^cf^	19.4 ± 0.9^ce^	23.4 ± 1.0^cd^
	500	19.2 ± 0.8	17.0 ± 1.0^ce^	15.0 ± 0.9^cf^	13.4 ± 1.0^cf^	16.2 ± 1.1^cf^	20.4 ± 0.9^cf^
Sukkari	250	20.4 ± 1.0	18 ± 0.7^cd^	18.4 ± 0.8^ce^	17.2 ± 0.7^cf^	20.4 ± 0.8^cf^	23.2 ± 0.9^cd^
	500	20.2 ± 0.9	17.2 ± 1.0^cd^	15.4 ± 0.9^cf^	14.6 ± 0.7^cf^	16.6 ± 1.1^cf^	21.0 ± 1.0^ce^

^a^ *p* < 0.05 vs. diazepam, ^b^ *p* < 0.01 vs. diazepam, ^c^ *p* < 0.001 vs. diazepam, ^d^ *p* < 0.05 vs. control, ^e^ *p* < 0.01 vs. control, ^f^ *p* < 0.001 vs. control

### Results of acetic acid induced writhing

All three date extracts significantly reduced acetic acid induced writhing in test mice as compared to the control. Diclofenac sodium, used as the positive control in this study showed strong analgesic activity (Table 4).

**Table 4:** Effects of three date extracts on acetic acid induced writhing in mice

Treatment (*n* = 5)	Dose (mg/kg)	Number of writhing
Control (1 % Tween 80 in water)	10 ml/kg	33.0 ± 1.0
Diclofenac sodium	25	9.4 ± 0.5^d^
Ajwah	250	23.0 ± 0.4^cd^
	500	21.0 ± 0.6^cd^
Safawy	250	24.0 ± 0.4^cd^
	500	22.0 ± 0.5^cd^
Sukkari	250	25.0 ± 0.7^cd^
	500	23.0 ± 0.6^cd^

^a^ *p* < 0.05 vs. diclofenac sodium, ^b^ *p* < 0.01 vs. diclofenac sodium, ^c^ *p* < 0.001 vs. diclofenac sodium, ^d^ *p* < 0.001 vs. control

### Results of hot plate test

The response time in test mice was extended by all the three date extracts and morphine as compared to the control and the results were statistically significant. Maximum effect was observed one hour after the treatment, which gradually faded at the end of the experiment (2 h) (Table 5).

**Table 5:** Effects of three date extracts on hot plate test in mice

Treatment (*n* = 5)	Dose (mg/kg)	Response time (sec)				
		0 min	30 min	60 min	90 min	120 min
Control	10 ml/kg	4.6 ± 0.13	4.5 ± 0.26	4.5 ± 0.18	4.2 ± 0.32	4.4 ± 0.15
Morphine	5	4.7 ± 0.15	8.9 ± 0.16^f^	11.4 ± 0.40^f^	11.0 ± 0.36^f^	8.7 ± 0.20^f^
Ajwah	250	4.3 ± 0.10	5.7 ± 0.24^cf^	5.9 ± 0.14^cf^	5.0 ± 0.10^ce^	4.4 ± 0.15^c^
	500	4.3 ± 0.1	5.9 ± 0.27^cf^	7.0 ± 0.19^cf^	6.6 ± 0.20^cf^	5.2 ± 0.10^cd^
Safawy	250	4.6 ± 0.15	5.7 ± 0.17^cf^	6.0 ± 0.13^cf^	5.3 ± 0.19^cf^	4.3 ± 0.14^c^
	500	4.6 ± 0.15	6.6 ± 0.20^cf^	7.3 ± 0.14^cf^	6.5 ± 0.21^cf^	4.5 ± 0.20^c^
Sukkari	250	4.2 ± 0.12	5.2 ± 0.12^c^	5.6 ± 0.15^cf^	4.9 ± 0.23^c^	4.3 ± 0.17^c^
	500	4.4 ± 0.14	5.9 ± 0.17^cf^	6.9 ± 0.15^cf^	5.8 ± 0.12^cf^	4.5 ± 0.17^cf^

^a^ *p* < 0.05 vs. morphine, ^b^ *p* < 0.01 vs. morphine, ^c^ *p* < 0.001 vs. morphine, ^d^ *p* < 0.05 vs. control, ^e^ *p* < 0.01 vs. control, ^f^ *p* < 0.001 vs. control

### Results of HPLC analysis

**Fig. 2:**
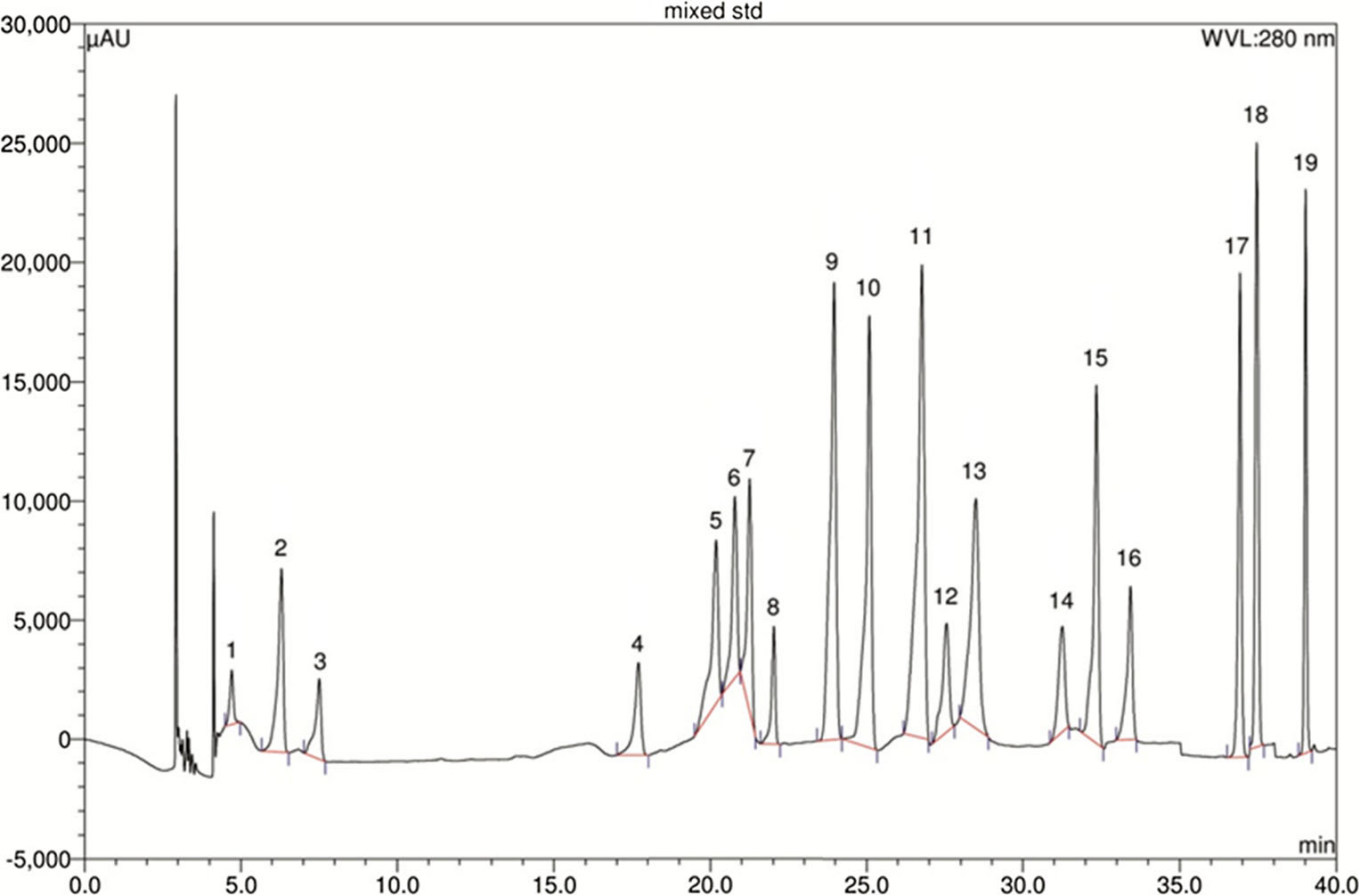
HPLC chromatogram of a standard mixture of polyphenolic compounds. Peaks 1: arbutin; 2: gallic acid; 3: hydroquinone; 4: (+)-catechin; 5: vanillic acid; 6: caffeic acid; 7: syringic acid; 8: (−)-epicatechin; 9: vanillin; 10: p-coumaric acid; 11: *trans*-ferulic acid; 12: rutin; 13: ellagic acid; 14: benzoic acid; 15: rosmarinic acid; 16: myricetin; 17: quercetin; 18: *trans*-cinnamic acid; 19: kaempferol

**Fig. 3:**
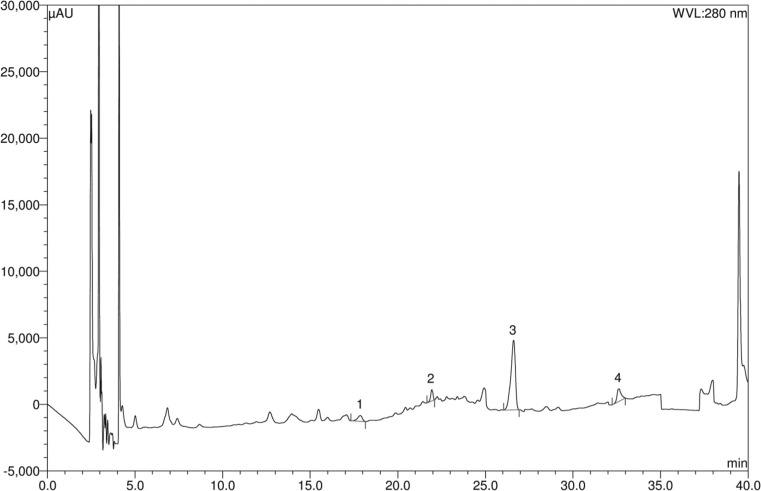
HPLC chromatogram of Ajwah date extract. Peaks 1: (+)-catechin; 2: (−)-epicatechin; 3: *trans*-ferulic acid; 4: rosmarinic acid

**Fig. 4:**
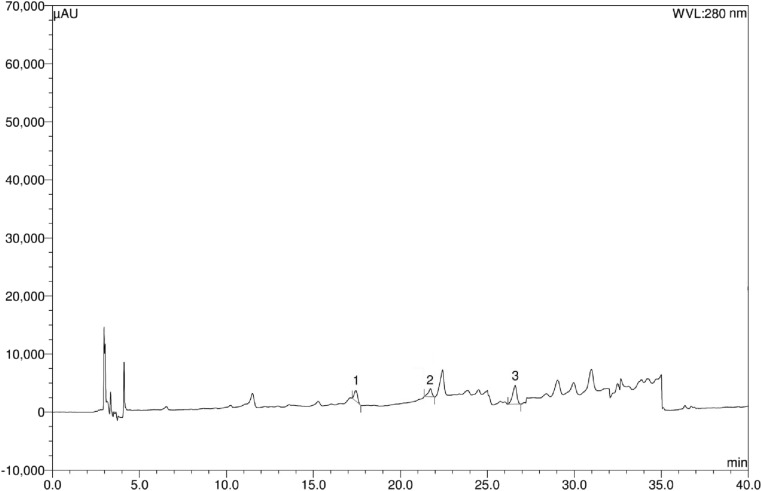
HPLC chromatogram of safawy date extract. Peaks 1: (+)-catechin; 2: (−)-epicatechin; 3: *trans*-ferulic acid

**Fig. 5:**
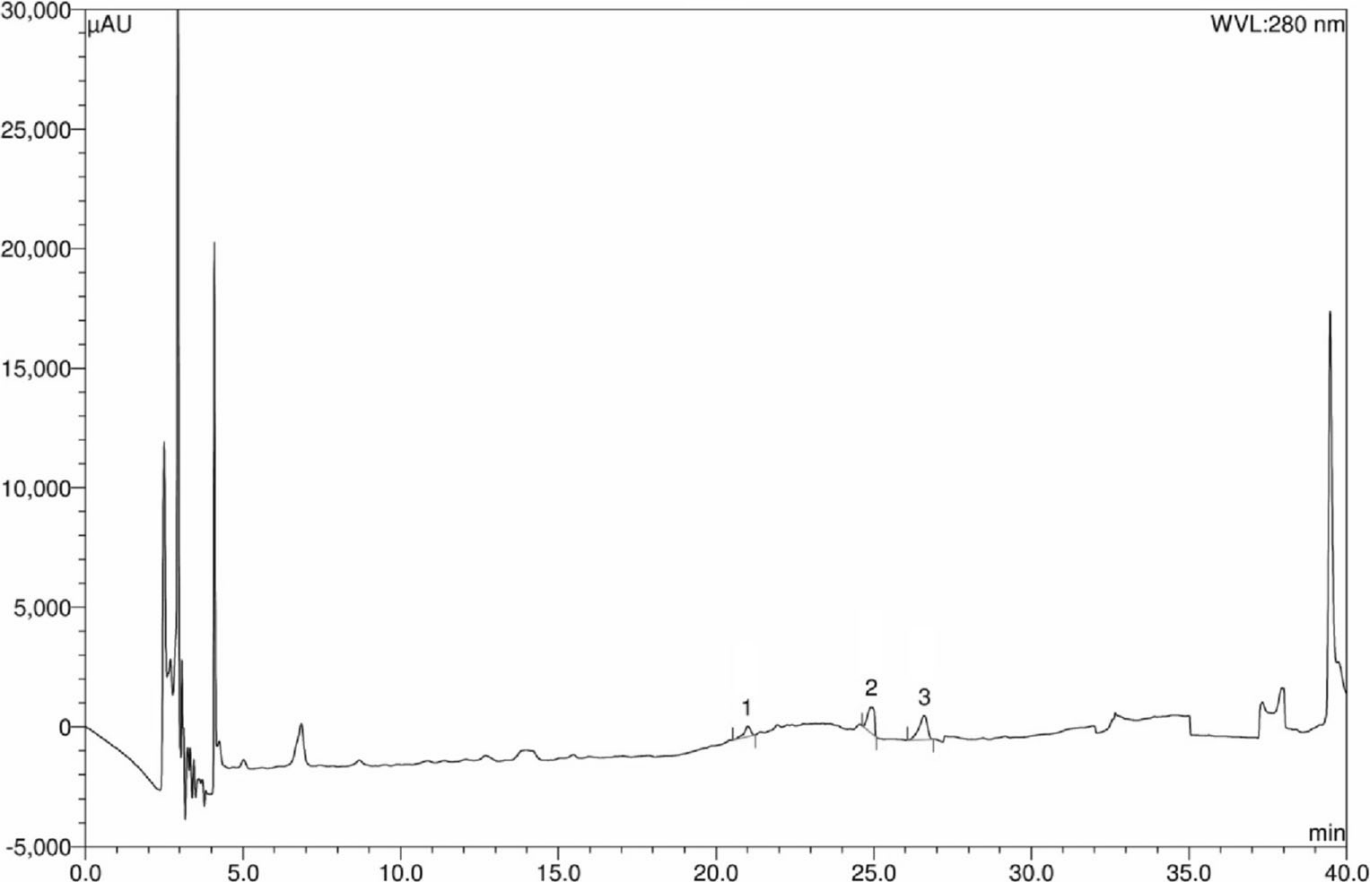
HPLC chromatogram of Sukkari date extract. Peaks 1: caffeic acid; 2: *p*-coumaric acid; 3: *trans*-ferulic acid

Results of HPLC analysis of the standards and three date cultivars under investigation are presented in Figs. 2, 3, 4 and 5 and Table 6. All the three date cultivars showed the presence of *trans*-ferulic acid with its highest content in Ajwah. Among other phenolic components, (+)-catechin and (−)-epicatechin were present in Ajwah and Safawy but not in Sukkari. In contrast, caffeic acid and *p*-coumaric acid were present only in Sukkari. Rosmarinic acid was only present in Ajwah.

**Table 6:** Contents of polyphenolic compounds in three date extracts

Polyphenolic compound	Content in mg/100 g of dry extract*(% RSD)		
	Ajwah	Safawy	Sukkari
*trans*-Ferulic acid	11.70 (0.18)	5.01 (0.06)	2.28 (0.06)
(+)-Catechin	14.67 (0.29)	42.25 (0.57)	–
(−)-Epicatechin	9.15 (0.11)	21.93 (0.34)	–
Rosmarinic acid	3.73 (0.04)	–	–
Caffeic acid	–	–	3.11 (0.09)
*p*-Coumaric acid	–	–	1.37 (0.05)

*n = 5; *RSD* Relative standard deviation

## Discussion

The fruits of date palm have a long history of its use in traditional medicine. While date palm is reported to be used in headache, recent study suggests that the fruits have cerebroprotective activity in mice suffering from cerebral ischemia (Kalantaripour et al. 2012). It was also found to exhibit neuroprotective activity in mice with ischemia induced bilateral common carotid artery occlusion (Pujari et al. 2011). Presence of flavonoids, sterols and ascorbic acid was credited for the observed ameliorating effect. Present investigation was done to evaluate neuropharmacological and antinociceptive effects of three cultivars of date palm, namely Ajwah, Safawy, and Sukkari. Extended period of sleeping by the test mice in acute toxicity test suggests that the effect was not a ‘post lunch dip’ which might occur with high sugar content of date palm extracts. All these extracts showed an increase in the pentobarbitone induced sleeping time in mice. Pentobarbitone is a barbiturate type sedative and hypnotic agent, which acts through allosteric modification of GABA receptor resulting in postsynaptic inhibition (ffrench-Mullen et al. 1993). Neuroactive agents, depending on their stimulating or depressing effect, can increase or decrease the duration of pentobarbitone induced sleep in test animal. In our present study, all the date extracts decreased the latency for the onset of sleep, as well as increased the duration of sleep indicating that the extracts might have some sedative effect on CNS. Open field and hole board tests are important but simple ways of determining CNS effect of any agent (Takagi et al. 1971; Uddin et al. 2006). Results of the present investigation shows a decrease in locomotor activity in test mice treated with date extracts suggesting that the date extracts might have decreased CNS activity in test mice. In both open field and hole board test, the effect was highest with Ajwah extract. Present investigation suggests a relaxing effect in the test mice treated with date extracts. It is well established that antioxidants play an important role in reducing oxidative stress in brain and provide neuroprotective effect (Giacalone et al. 2011; Mohamadin et al. 2010; Sheikh and Mohamadin 2012; Wang et al. 2006). In our present investigation, two important flavanols, namely, (+)-catechin, and (−)-epicatechin were detected in Ajwah and Safawy extracts. Neuroprotective effect of these two flavonols is well established and the mechanism of action is believed to be their antioxidant activity and beneficial actions on brain cells which include positive effects on mood (Mandel and Youdim 2004; Nehlig 2013). Although neuroprotection by hydroxycinnamic acids can be much less as compared to catechins, the effect of *trans*-ferulic acid cannot be ruled out since it is reported to exert neuroprotective effect in *in-vivo* and *in-vitro* tests and its antioxidant capacity might be the contributing factor for such activity (Cheng et al. 2008; Luo and Sun 2011; Wu et al. 2014). The traditional use of date plam in headache prompted us to test the extracts for antinociceptive activity. Decrease in the writhing in acetic acid induced writhing test suggests that date extracts can show analgesia through peripheral mechanism of pain inhibition, i.e., block inflammatory pathway of pain sensation through the inhibition of prostaglandin synthesis (Murata et al. 1997). This is in agreement with previous finding in which methanol extract of Zaghlool dates showed anti-inflammatory activity in rat model (Mohamed and Al-Okabi 2004). An increase in the response time in hot plate test further suggests that the observed analgesia might also involve centrally acting mechanism (Wigdor and Wilcox 1987). In different studies, *trans*-ferulic acid has showed analgesic activity in thermal hyperalgesia, acetic acid induced writhing and mechanical allodynia tests in mice (Lv et al. 2013; Ozaki 1992). Thus, *trans*-ferulic acid could be credited to some extent for the observed analgesic activity of the date extracts. In addition, more potent activity of Ajwah compared to the two other date cultivars might be due to the higher content of *trans*-ferulic acid in Ajwah. (+)-Catechin, and (−)-epicatechin, detected in Ajwah and Safawy extracts are also reported to show anti-inflammatory activity in various *in-vivo* and *in-vitro* models including inhibition of NO production and LPS-induced prostaglandin E2 release (García et al. 2013; Wang and Cao 2014; Yang et al. 2015).

## Conclusion

Present investigation suggests that Ajwah, Safawy and Sukkari cultivars of date palm have some degree of relaxing effect on the brain. It is possible that these extracts reduce CNS activity resulting in decreased locomotor activity in test mice. The extracts also produced analgesic activity in test mice supporting its use in headache in traditional medicine. However, in all cases, the effect was not as strong as that of the positive control, indicating a moderate level of neuropharmacological and analgesic activity, and thus could be of interest for producing mild relaxing effect on the brain. The effects were similar with all the three date cultivars, but relatively stronger with Ajwah dates.

## Electronic supplementary material

ESM 1 (JPEG 251 kb)

ESM 2 (JPEG 419 kb)

ESM 3 (JPEG 350 kb)
