# Supplementary figures and images for: Comparative study of neuropharmacological, analgesic properties and phenolic profile of Ajwah, Safawy and Sukkari cultivars of date palm (Phoenix dactylifera)
Source: Orient Pharm Exp Med. 2016 Aug 16;16(3):175–83. doi: 10.1007/s13596-016-0239-5 (PMC5040737; doi:10.1007/s13596-016-0239-5)

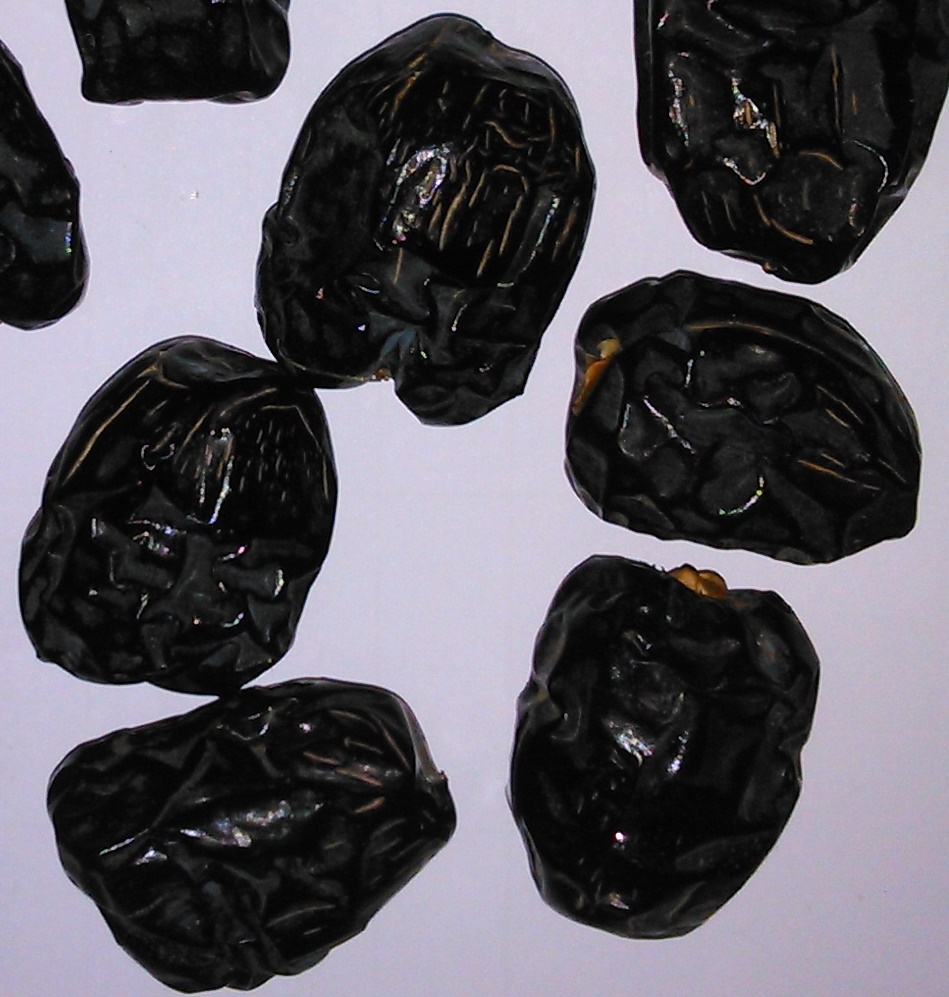

Supplement: Supplementary file 1 — (JPEG 251 kb) [file 13596_2016_239_MOESM1_ESM.jpg]

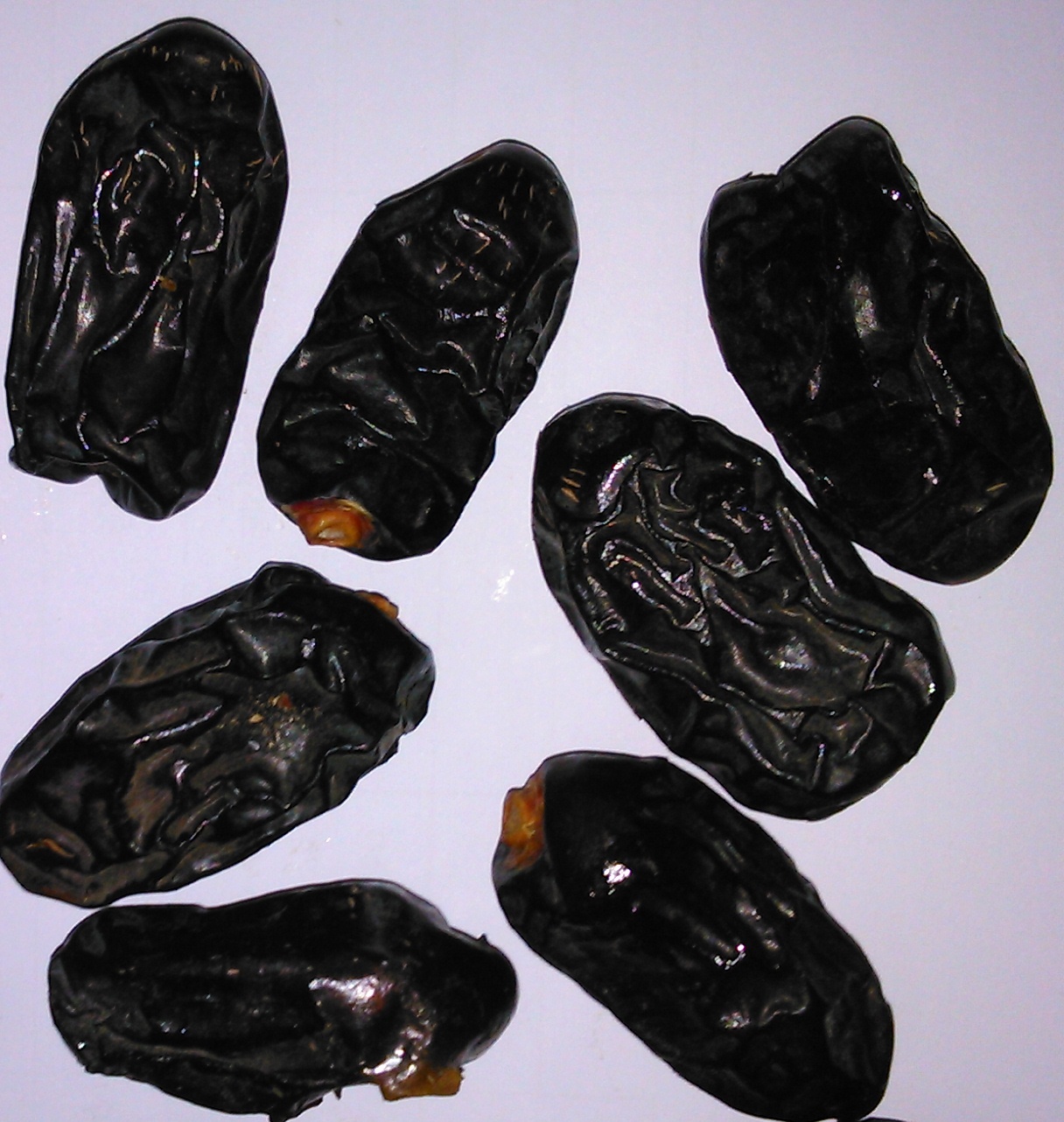

Supplement: Supplementary file 2 — (JPEG 419 kb) [file 13596_2016_239_MOESM2_ESM.jpg]

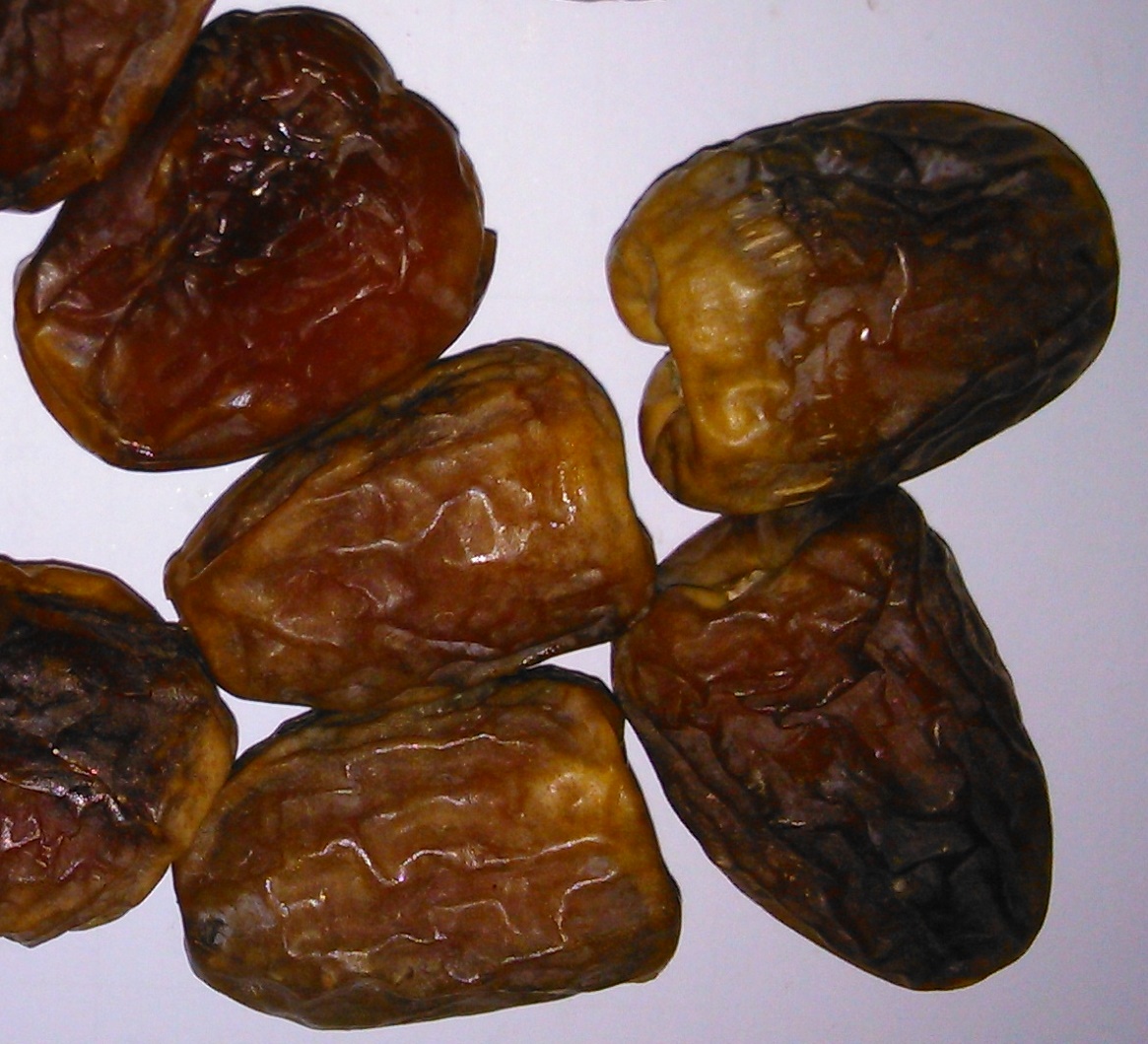

Supplement: Supplementary file 3 — (JPEG 350 kb) [file 13596_2016_239_MOESM3_ESM.jpg]
